# Resin Use by Stingless Bees: A Review

**DOI:** 10.3390/insects12080719

**Published:** 2021-08-11

**Authors:** Maggie Shanahan, Marla Spivak

**Affiliations:** Department of Entomology, University of Minnesota, 219 Hodson Hall, 1980 Folwell Ave, St. Paul, MN 55108, USA; spiva001@umn.edu

**Keywords:** resin, propolis, stingless bees, Meliponini, colony health, microbiome

## Abstract

**Simple Summary:**

Bees, ants, and other insects harvest antimicrobial resins from plants and use this material for a variety of purposes, from nest construction to defense against predators and pathogens. Resin use is thought to have facilitated the evolution of sociality in stingless bees, and today, resin use remains fundamentally important for stingless bee colony function. Most species use resin to build brood comb, storage pots for honey and pollen, and various protective structures within the nest. Many also use resin to protect their nests from predators, fortifying nest entrances with a barrier of sticky resin droplets or applying resin directly to would-be invaders. For some species, the presence of resin inside the nest space can also influence the physical properties of the bees themselves, enriching the chemical composition of the outermost layer of their exoskeleton, and possibly shaping the communities of bacteria and fungi that are found on the bees, and in their nests. This article brings together studies from a variety of fields to illustrate the importance of resin use for stingless bee colony function and conservation, and to point towards areas of future research.

**Abstract:**

Stingless bees (Meliponini) are highly social bees that are native to tropical and sub-tropical ecosystems. Resin use is vital to many aspects of stingless bee colony function. Stingless bees use resin to build essential nest structures, repel predators, and kill would-be invaders. Furthermore, resin-derived compounds have been found to enrich the cuticular chemical profiles of many stingless bee species, and resin may play an important role in shaping the microbial communities associated with stingless bees and their nests. Despite its importance for colony function, previous reviews of resin use by stingless bees are lacking. This topic grows increasingly urgent as changes in beekeeping and land use practices occur, potentially diminishing stingless bees’ ability to incorporate resin into the nest environment. In this article, we review existing literature on resin use by stingless bees and discuss potential areas of future research.

## 1. Introduction

Stingless bees (Meliponini) are highly social bees that are native to tropical and sub-tropical ecosystems. With approximately 550 species known to science, stingless bees comprise the largest and most diverse group of corbiculate bees (Euglossini, Apini, Bombini, and Meliponini). They represent approximately 70% of all eusocial bee species [1], and exhibit a dizzying diversity of morphologies, behaviors, and life histories. Their geographic distribution spans five continents, and their colonies can range in size from a few hundred to many thousands of individuals. Their nesting habits vary widely, with some species nesting in tree cavities, others nesting inside active termite or ant nests, and still others building subterranean nests up to three meters underground [2]. Humans have been in relationship with stingless bees for millennia through the practice of stingless beekeeping, or meliponiculture [3,4,5]. In fact, stingless bee research often draws from the local ecological knowledge that stingless beekeepers from indigenous and rural communities have cultivated for generations. This includes information on the myriad medicinal uses for the resinous materials that beekeepers harvest from stingless bee nests [6,7,8,9].

Stingless bees collect the sticky resins that plants secrete and use this material for a variety of purposes. Most species use resin to build essential nest structures such as brood comb, storage pots for honey and pollen, and various protective structures [2,10]. For many species, resin is also an important part of nest defense; stingless bees use resin to build barriers, trap predators, and kill would-be invaders [11,12,13,14,15]. For some species, the presence of resin inside the nest space can also influence the physical properties of the bees themselves and their microbial associates. Resin-derived compounds have been found to enrich the cuticular chemical profiles of many stingless bee species [16], and resin may play an important role in shaping the microbial communities associated with stingless bees and their nests [17,18,19,20].

Despite the importance of resin use for stingless bee colony function, previous reviews of this topic are lacking. In part, our understanding of resin use is limited because less than half of all meliponine nests have been described by Western science [10]. Of these, only a small number of species have been studied intensively, and few studies have focused specifically on resin use (except see studies by the Leonhardt group, cited below). The information we do have is difficult to generalize across species because stingless bees are highly diverse, and resin use is a particularly variable trait. Lastly, as is the case for living systems throughout the world, scientific literature represents only a limited portion of human knowledge of stingless bees. Though there have been numerous recent efforts to account for indigenous and local ecological knowledge of stingless bees [5,8,21], Western science has historically excluded these knowledge systems, so a review of the existing literature is limited in scope.

In spite of these challenges, this review brings together research from disparate fields (e.g., natural history, chemical ecology, microbiology) to examine resin use in stingless bees. Taken together, these studies highlight the centrality of resin use to stingless bee colony function. In the following sections, we review existing literature on the role of resin in stingless bee nest construction and defense, discuss resin foraging and resin handling by stingless bees, and review studies on the effects of resin on bees’ cuticular chemical profiles and their microbial associates. Finally, we point to gaps in knowledge that warrant further study.

## 2. What Is Resin?

The chemistry, evolution, ecology, and ethnobotany of plant resins has been reviewed by Langenheim [22]. Plants secrete resin from buds, wounds, fruits, and flowers to defend themselves from herbivores and microorganisms, and, in some cases, to attract pollinators and seed dispersers [22,23,24,25]. Resin can trap, immobilize, or deter predators, disinfect wound sites, and help to guard against the proliferation of endophytic fungi [22]. This versatile material is chemically complex. It consists of lipid-soluble mixtures of volatile and non-volatile phenolic compounds (e.g., flavonoids, aromatic acids, and benzopyranes) and terpenoids (e.g., mono-, di-, and sequiterpenes) that possess a variety of anti-inflammatory, antifungal, antibacterial, and antiviral properties [22]. The specific chemical composition of resin varies between plant species, and can even vary between individuals of the same species [26]. Predators and pathogens are limited in their ability to evolve resistance to the complex and variable mixture of bioactive compounds that resin contains.

A wide variety of animals—from humans to coatis to wood ants to bees—harvest resin from plants and use this resource as a medicine, defense, and building material [22,25,27,28,29]. Many bee species use plant resins in nest construction; of these, the majority belong to the families Megachilidae and Apidae. In fact, with the exception of bumblebees (Bombini), almost all corbiculate bees harvest and make use of plant resins [30]. Resin use by honey bees has received increasing attention in recent years (reviewed by Simone-Finstrom and Spivak [31], Simone-Finstrom et al. [32], and Mountford-McAuley [33]). In stingless bees, resin use is even more extensive; many stingless bee species collect resin in copious amounts and use it to support multiple aspects of colony function.

## 3. Resin Use by Stingless Bees

### 3.1. Nest Construction

Nest construction strategies vary widely across stingless bee species, with different species building nests in different spaces. Some species build exposed nests adhered to tree branches, others make use of existing cavities including hollow trees, termite nests, or electric light posts, and others build subterranean nests deep underground [1,2,34]. Nest construction materials range from fecal matter to soil to human-made products such as wet paint and adhesives [10]. Though nest construction varies both across and within species [35], almost all stingless bees use resin in some part of their nest.

Resin is an effective construction material for several reasons. Resins are malleable when secreted but harden over time, so they can be shaped to build durable structures [22]. They are also water insoluble, so they can be used to create waterproof nest spaces and water-tight storage pots [29,36]. Lastly, the antimicrobial properties that resins possess may help regulate the microbial communities found inside stingless bee nests, preventing food spoilage and pathogen attack [29]. In fact, the use of antimicrobial resins in nest construction may have been central to the evolution of sociality in stingless bees.

For many insects and other organisms, managing microbial communities is a key part of building and maintaining a successful nest. These efforts are particularly important in tropical environments, where conditions favor the proliferation of microbes, and in social insect societies, where the risk of disease transmission is increased due to large numbers of genetically similar individuals living in close proximity [29,37]. Many insects use antimicrobial compounds to prevent the spoilage of food and the spread of pathogens. These compounds can be self-produced (e.g., many stinging insects apply antimicrobial venom to their cuticle and nests [38,39]), symbiont-produced (e.g., beneficial microbes secrete antimicrobial compounds that prevent the spoilage of food stores [40]), or environmentally acquired (e.g., bees, ants, and other insects bring foreign materials—such as antimicrobial resin—into their nests [28,41]). Since the use of foreign materials allows for new forms of nest construction, this evolutionary adaptation is thought to have facilitated a massive range expansion and diversification for bees [42]. Because resins help preserve food stores and enable the construction and defense of resource-rich nest spaces, resin use, specifically, is thought to have facilitated the social evolution of stingless bees in tropical ecosystems [29] (p. 388) and the subsequent diversification of stingless bee species [43]. A molecular phylogeny constructed by Rasmussen and Camargo [35] supports this hypothesis, indicating that ancestral *Trigona* species likely used resin to build their nests. Today, resin use persists as a crucial component of nest construction for stingless bees.

**Figure 1:**
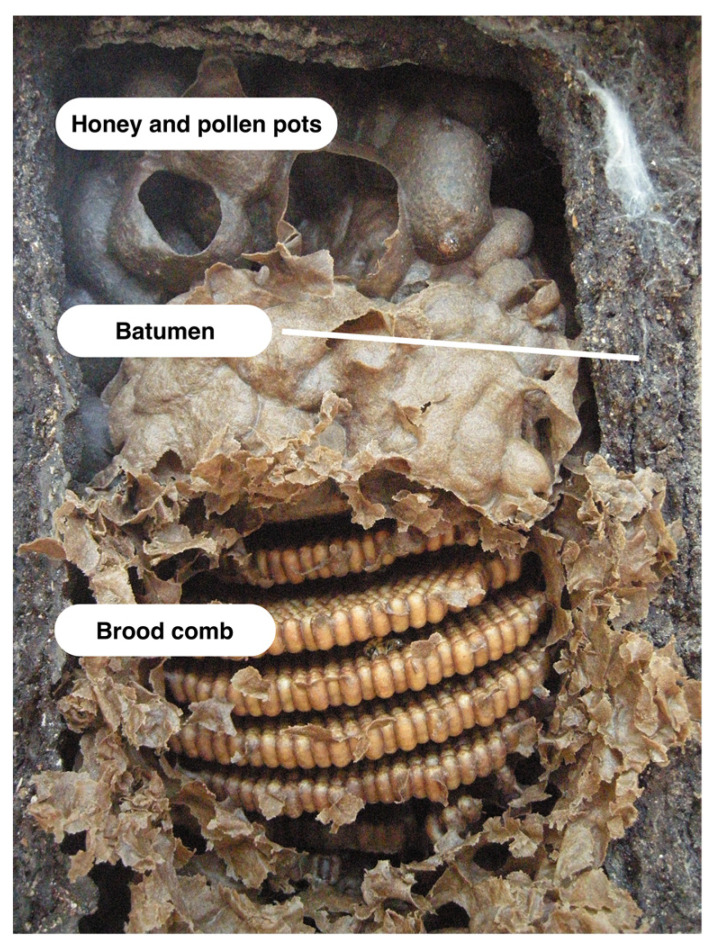
Nest structures such as brood comb and honey and pollen pots are made of cerumen, a mixture of wax and resin. The batumen is a wall-like structure that surrounds and protects many stingless bee nests; it is often made of resin. Photo by Miguel Angel Guzmán Díaz.

Most stingless bees mix resin with wax to produce cerumen, which is the material they use to build brood combs, honey and pollen pots, and various other structures inside the nest (except see *Schwarzula* sp. [44] and *Trigona australis* [45]) (Figure 1). Because it contains both wax and resin, cerumen has sometimes been equated with honey bee propolis [46]. However, cerumen serves as the primary construction material within the stingless bee hive, so it is actually closer in function to beeswax, though it differs from beeswax in several important ways. Rather than forming part of a permanent comb structure, cerumen is continuously reworked and recycled within the nest. The physical properties of cerumen can vary. This is likely due, at least in part, to the variable proportions of wax and resin the material contains. Cerumen can be soft, flexible, and light brown in color (possibly containing more wax and less resin) or rigid, brittle, and dark brown or black in color (possibly containing less wax and more resin) [47]. Although there has not yet been a comparative study of cerumen characteristics across species, Roubik [10] noted that the cerumen produced by certain small stingless bees (e.g., genera *Hypotrigona, Trigonisca, Schwarzula,* and *Plebeia*) contains little to no resin, and is closer to pure wax. *Schwarzula* sp. appear to use no resin at all, instead farming scale insects within the nest cavity and mixing their wax with self-produced wax to form a cerumen equivalent [44]. At the other end of the spectrum, the resin content of cerumen in some species can surpass 40% [2]. The factors that influence the amount of resin that different stingless bee species incorporate in cerumen—and in other parts of the nest—are not yet understood. Blomquist et al. [48] suggested that excluding resin may help some species cope with the high temperatures in the spaces where they nest, but this hypothesis has yet to be confirmed.

In addition to using resin to produce cerumen, stingless bees incorporate resin into the nest environment in the form of deposit-resins, propolis, and geopropolis (defined below), and in structures such as the nest entrance and batumen (Table 1). These terms are often conflated in the literature, with propolis being used as a catch-all to describe any resinous material inside the nest, aside from cerumen. However, it is useful to distinguish between these terms, since a single nest may contain multiple types of resin-rich materials and resin-based structures, each serving a different purpose.

Deposit-resins, also referred to as resin deposits or viscous propolis deposits [10,49,50], are resin caches located on the nest floor or walls [51]. For some species, these caches serve as temporary storage where resins accumulate until they can be incorporated into other nest structures or used for defensive purposes (e.g., *Trigona* (*Trigona*) *p. pallens* [52]; *Tetragonisca angustula* (Latreille) and *Plebeia* spp. [29,50]). Unlike most resin, which hardens upon contact with air, deposit-resins remain viscous for a prolonged period of time. This property could have to do with the resin source; deposit-resins may contain a greater proportion of floral resins, which are slow to harden [53]. Alternatively, or additionally, the prolonged viscosity of deposit-resins could result from chemical processing. While definitive research on this topic is lacking, a comparative analysis of the morphology of head salivary glands and intramandibular glands of bees of various ages suggests that *Plebeia emerina* workers modify deposit-resins using secretions, which might help to maintain their viscosity [50].

**Figure 2:**
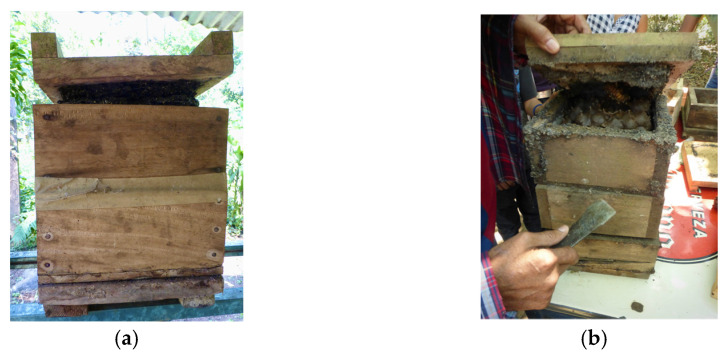
(**a**) Even in wooden box hives, many colonies seal cracks with a thick layer of propolis. (**b**) To access these colonies, beekeepers often use a hive tool to pry the lid from the hive body; excess propolis is sometimes lost in this process.

Propolis refers to the resins that stingless bees bring back to the nest and mix with small amounts of salivary gland secretions and, purportedly, wax [49]. Numerous studies report that stingless bee propolis is more chemically diverse than honey bee propolis (reviewed by Popova et al. [9]). As with honey bees, many stingless bee species use propolis to seal cracks and crevices throughout the nest. For colonies managed in box hives, bees often seal cracks with a layer of propolis so thick that beekeepers must pry the lid from the hive body in order to access the nest (Figure 2). Though most studies state that propolis contains wax, the extent to which stingless bees incorporate wax in propolis is unclear. The amount of wax in *A. mellifera* propolis is known to be highly variable, and reports of wax content often lack precision [54]. In a detailed study of stingless bee resin handling, Gastauer et al. [55] observed resin deposition in six bee species, but noted no mixing of resin with wax. It is possible that propolis produced by difference species and for different purposes could contain variable amounts of wax, and in some cases no wax at all, but this has yet to be verified.

Some stingless bees use geopropolis in place of pure propolis. Geopropolis is a mixture of plant resins and soil. This mixture can consist of up to 90% soil, silt, and sand particles [56]. It is less malleable than pure propolis, but serves a similar function inside the nest [57]. Some studies use the terms propolis and geopropolis interchangeably, or state that geopropolis is the propolis of stingless bees [58], but these are actually distinct materials, differentiated by the presence or absence of soil [9].

Propolis and geopropolis are often incorporated into other nest structures such as the nest entrance and batumen. Nest entrances commonly consist of hardened resin tubes, which can extend both inside and outside the nest. Internally, these convoluted maze-like structures are often designed to thwart enemy intruders [10]. The batumen, also called the external involucrum [59], is a wall-like structure that many stingless bee species build to separate the inner nest environment from the external world. This structure is usually made of resin and can also include mud, seeds, wood, feces, and other materials [10]. Batumen construction is a variable trait among stingless bee species. Some species construct sturdy batumen walls measuring up to 10 cm in thickness (e.g., *Melipona* spp. [10]); others build no batumen at all (e.g., *Hypotrigona* and *Trigonisca* spp. [47]). When present, the batumen can take many forms. In one comparative study of stingless bee nest architecture, Wille and Michener [47] described several batumen types, and noted their presence or absence for 145 stingless bee species found in Costa Rica. According to this study, *exposed batumen* is a hard outer layer that surrounds and protects exposed or partially exposed nests. *Batumen plates* are sturdy plates that surround and protect nests within a cavity, allowing the bees to adjust the cavity size to suit the needs of the colony (e.g., genera *Melipona* and *Cephalotrigona; Meliponula bocandei*) [10]. *Lining batumen* is a thin, continuous resinous lining, generally less than 2 mm in thickness, similar to the so-called propolis envelope that *A. mellifera* colonies use to coat the rough inner surfaces of the hollow tree cavities where they nest [60]. *Laminate batumen* consists of multiple layered sheets. The channels found in laminate batumen allow bees to move between layers, and may also facilitate air flow and water evaporation [10,47]. In addition to providing a protective shield, these various types of batumen may serve to waterproof the nest cavity and help control fungal growth [47].

### 3.2. Defense

Bees contend with a variety of predators and parasites. Some examples include lizards, spiders, ants, wasps, assassin bugs, beetles, phorid flies, and parasitic stingless bees from the genus *Lestrimelitta* [11,29]. Since stingless bees are unable to sting, they rely on a variety of other strategies to defend their nests. Defensive strategies vary across species and include such behaviors as hiding, building cryptic nests, biting, and burrowing in hair. Stingless bees also employ resin in a variety of ways to deter, trap, and kill predators and parasites. Here, we categorize resin-based defenses in two groups: (1) structural defenses, where bees build resinous structures or add fresh resin to existing structures to prevent invasion, and (2) direct defenses, where bees apply resin to the bodies of their enemies or to their own bodies to defend their nests.

#### 3.2.1. Structural Defenses

**Figure 3:**
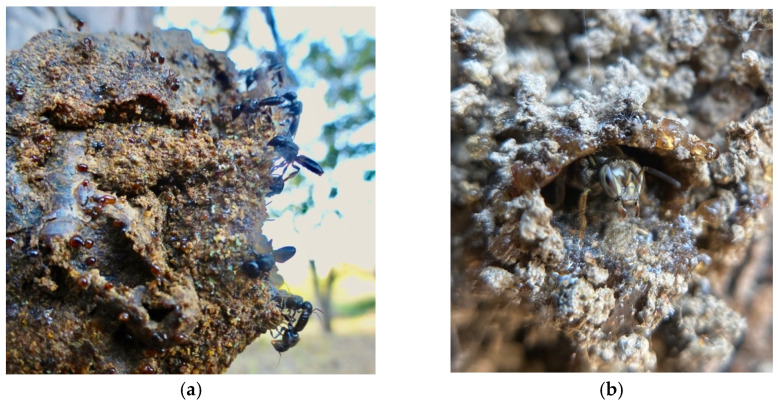
Many stingless bee species surround their nest entrances with (**a**) a barrier of resin droplets (**b**) or a continuous layer of resin to repel and trap would-be intruders. Photos by Héctor Morales Urbina.

Many stingless bee species (e.g., genera *Lepidotrigona, Scaura, Tetragona, Tetragonula,* and *Trigonisca*) fortify their nest entrances with a barrier of fresh resin droplets [10] (Figure 3). This sticky material serves as a defense that is both mechanical and chemical in nature (reviewed by Leonhardt [16]). The terpenoid compounds commonly found in resin repel many predators [10,14,22,61]. The predators (largely ants) that do attempt to advance across the resin droplets often become trapped in the sticky material [62], and are only able to breech the barrier when they use the bodies of other ants to bridge the so-called resin “moat” [2,63,64]. Over time, the resin droplets harden, their adhesive and repellant properties likely diminish, and fresh stores must be applied [62,65]. For some species (e.g., *Trigona cilipes, Tetragonilla collina*, and related species), the continuous application of fresh resin results in long, slender entrance tubes [10]. In the case of one remarkable species, nest entrance resin produces a dazzling architectural effect. The minute, tear-drinking stingless bee *Pariotrigona klossi* (Schwarz) builds a nest entrance consisting of dozens of tubelets that branch like coral. Each tubelet is adorned with strings of clear resin beads which together resemble the “quartz pendants of a chandelier” [63]. For invading ants, this resinous terrain is difficult to navigate when hardened, likely impassable when fresh, and may also be visually disorienting, further deterring ant attack.

Some stingless bees also use resin and cerumen pieces to barricade the nest entrance at night, or when disturbed (e.g., *Meliplebeia tanganyilcae medionigra* (Cockerell) and *Plebeiella lendliana* (Fries) [10,66]. Some species (e.g., *Melipona panamica, Melipona flavolineata*, and other *Melipona* species) even keep a designated resin ball on hand for this purpose [12]. When the colony is under attack, the bees roll the hardened resin ball into place and use fresh resin to fasten it to the entrance to prevent invaders from breaching the nest. Over time, discarded resin balls accumulate near the internal entrances of these nests [10,12].

Stingless bees that live in active termite or ant nests often surround their cavities with a full defensive resin barrier. This allows them to inhabit otherwise hostile environments. For instance, when some myrmecophilous stingless bees (e.g., *Trigona moorei*) initiate a nest, they begin by building a provisional batumen structure to establish ant-free spaces. They then expand this resinous shield as they burrow deeper into the ants’ nest [67]. A similar behavior can be seen in *Scaura latitarsus*; these bees form their nest by excavating a cavity in an active termite nest and lining that cavity with a continuous batumen shell [29].

#### 3.2.2. Direct Defenses

In addition to using resin in nest structures to prevent invasion, many species also apply resin directly to perceived threats. This behavior has been referred to as “resin daubing” (see detailed description of *Austroplebeia australis* nest defense by Halcroft et al. [11]), and it can lead to the immobilization or total mummification of predators [13]. When certain species sense a threat, defending bees harvest resin and cerumen from deposit-resins and/or other parts of the nest, carrying these materials in their mandibles and corbiculae [11]. They then attack would-be invaders outside the nest (e.g., plastering resin to human hair) [2], or trap and mummify intruders within the nest (e.g., immobilizing parasitic fly pupae, ants, and various types of beetles) [2,13,14,68,69]. Curiously, some stingless bee species also use “resin pellets” to kill virgin queens from their own colonies when these are in excess [70].

Stingless bees do not just apply resin to the bodies of intruders; some species apply resin to their own bodies as well (see also Section 3.5: Cuticular chemical profile). Several stingless bee species have been observed leaving the nest with small amounts of both viscous and hardened resin in their corbiculae [71,72]. While soft, sticky resin can be used to entangle would-be invaders, the reason for carrying hardened resin is not entirely clear. In *Melipona subnitida*, Harano et al. [72] observed that 11% of worker bees leaving the nest carried resin in their corbiculae under normal (i.e., undisturbed) conditions. About half of these carried soft, sticky resin loads, while the other half carried dry, hardened resin. When the nest was disturbed, the number of worker bees leaving the nest with resin increased to 90%, with a majority (80%) of these carrying hardened resin. Both resin bearers and nectar foragers were paint-marked, and their movements were monitored. The short flight duration for resin bearers suggested that they were circling the nest, rather than relocating resources to an alternative nest site in response to predator attack. The authors speculated that, because of its repellant properties, hardened resin may serve as a type of armor, deterring would-be predators from eating the resin bearers. Alternatively, the resin bearers may sacrifice themselves for the benefit of the colony; after eating one unpalatable resin bearer, a predator might be dissuaded from further predation. This is not the first account of stingless bees carrying visible amounts of resin on their bodies for a purpose other than resin-daubing. The cuticle of *Tetragonula carbonaria* is covered with resin, so that the whole body is sticky; a thin layer of resin has been observed on the legs, head, and thorax of *Tetragonsica angustula*, and *Trigona (Tetragonula) melanocephala* nectar foragers have been observed leaving the nest with resin in their corbiculae [71,73,74,75]. However, the study conducted by Harano et al. [72] provides the first detailed observation of bees carrying hardened resin on their bodies as part of an apparent mobilized defense, taking a piece of their nest with them for individual or collective protection.

Resin-based defenses can be triggered by both visual stimulation and chemical cues. In *Melipona flavolineata* (Friese), the head secretions and mandibular gland extract of the robber bee *Lestrimelitta limao* (Smith) elicited increased resin transport and the barricading of the nest entrance tube with hardened resin balls [12]. In *Tetragonilla collina*, resin foraging activity increased after nest entrances were damaged, and doubled after ant attack; worker bees used resin to elongate their entrance tubes and fortify them with a barrier of resin droplets [62].

Resins from different plant species are effective against different predators and pathogens, and stingless bees may select resins based on their functional properties. This means that access to diverse resin sources is important for stingless bee defense [15]. It is not yet clear whether stingless bees alter resin resource preferences in response to pressure from specific threats (i.e., collecting resins that are particularly effective in repelling small hive beetles in response to a small hive beetle attack). It is also unclear whether stingless bees use resin as a defense against bacterial or fungal pathogens (see also Section 3.6: Microbiota Associated with Stingless Bees). In *A. mellifera*, the presence of a propolis envelope has been found to decrease the severity of multiple brood diseases [32]. Furthermore, colonies increase resin collection when challenged with *Ascosphaera apis*, the causative agent of the larval disease chalkbrood. This suggests that honey bee colonies use resin to self-medicate in response to certain pathogens [32,76]. Similar behaviors may occur in stingless bees, and numerous studies indicate that stingless bee resin inhibits the growth of multiple microbes (reviewed by Bankova and Popova [77]). However, with the exception of the bacterium *Lysinibacillus sphaericus*, which causes broods to degenerate, there are no known examples of pathogenic microbes in stingless bee colonies (Heard 2016; as cited in [16]). Consequently, the antimicrobial activity of resin is generally tested against human pathogens, so its effect on microbes associated with stingless bee colonies is unknown.

### 3.3. Resin Foraging

Although resin is essential to many aspects of stingless bee nest construction and defense, little is known about how stingless bees obtain this vital resource. Some information on resin foraging can be gleaned from studies on general foraging behavior [78,79,80,81], but there are few studies that examine resin foraging in stingless bees specifically (except see [24,62,65]).

Resin foragers make up <10% of the foraging force for many species (e.g., *Tetragonula minangkabau, Heterotrigona itama, Trigonella moorei, Melipona bicolor bicolor, Trigona sapiens,* and *Trigona hockingsi*) [24,82,83], and are often outnumbered by pollen foragers (e.g., *Melipona bicolor schencki* (Gribodo), *Trigona iridipennis* (Smith), and *Melipona fasciculata* (Smith)) [78,81,84]. However, some species collect copious amounts of resin, with resin foragers outnumbering pollen foragers (e.g., *Melipona asilvai* [79]). For *Tetragonula carbonaria*, resin foragers can account for up to 50% of the foraging force [85]. For *Tetragonilla collina*, up to 90% of foragers have been observed returning with resin, likely during periods of nest construction [62]. This is in stark contrast to *A. mellifera*, where resin foragers make up only 1–3% of the foraging force [33].

A variety of environmental (e.g., temperature, light intensity, humidity, resource availability) and colony (e.g., population size, developmental stage) conditions influence resin foraging frequency at the colony level, and these factors have different effects on different species [86]. For example, for some species (e.g., *Trigona iridipennis, Melipona asilvai, Melipona bicolor schencki,* and *Melipona colimana*) resin foraging activity fluctuates seasonally, but for other species (e.g., *Melipona fasciculata*), resin foraging is constant throughout the year [78,79,81,84,87]. Seasonal changes in resin collection could be related to many variables, such as resource availability, fluctuating pathogen pressure, and colony developmental stage [81], but these are largely unexplored. For some species (e.g., *Melipona bicolor bicolor*), resin foraging increases with colony strength (as determined by comb diameter) [83]. For others, resin collection may be intense in the early stages of colony development and then taper off once the structural components of the nest are established [62]. For species that use resin-daubing or resin barriers as a form of defense, pathogen pressure can lead to increased resin foraging [62]. Finally, there is some evidence that certain species (e.g., *Plebeia emerina*) hoard resin stores, possibly in preparation for periods of resin scarcity, or in preparation for increased predator or parasite pressure [49].

Resin collection is primarily carried out by worker bees [88]. Curiously, Boongird and Michener [34] observed resin and pollen loads on the hind tibiae of male stingless bees from several species in Thailand (*Tetragonula fuscobalteata* (Cameron), *Tetragonula (Tetragonula) pagdeni* (Schwarz), *Tetragonula collina* (Smith), and *Heterotrigona (Tetrigona) apicalis* (Smith)). It is unclear whether and how resin-bearing males contribute to colony function, but since they were not seen depositing their loads in storage pots or on other nest structures, the authors concluded that male bees do not contribute significantly to resin foraging.

At an individual level, it is unclear what factors drive a forager to choose resin foraging over nectar or pollen foraging. In *Melipona beecheii*, Biesmeijer and Tóth [89] found that half of observed foragers specialized in just one resource throughout their foraging career, and the other half alternated between pollen, nectar, resin, and mud [89]. This result is consistent with Inoue et al. [82], who examined foraging behavior in three Sumatran stingless bee species (*Trigona (Tetragonula) minangkabau* (Sakagami and Inoue), *Trigona (Heterotrigona) itama* (Cockerell), and *Trigona (Trigonella) moorei* (Schwarz)) and found approximately 50% of foragers to be one-material specialists. In *A. mellifera*, foragers initiate resin collection when they detect a need for it inside the nest (e.g., by sensing a rough surface, crevice, or draft of cool air), and use the waggle dance to recruit additional resin foragers [90]. It is unclear whether stingless bees respond to similar stimuli, and whether and how they recruit other bees to collect resin.

When foraging, stingless bees use both visual and olfactory cues to discover and distinguish between resin sources. Specifically, they home in on particular combinations of volatile mono- and sesquiterpenes [26]. This sensory capacity allows stingless bees to discover new resin sources quickly, sometimes locating artificially induced tree wounds within a matter of minutes [65]. When certain resin sources are highly preferred, as occurs in the seed-dispersal mutualism between the Eucalypt tree *Corymbia torelliana* and the stingless bee *Tetragonula carbonaria*, even minor experimental modifications to a resin odor (i.e., changes in single mono- or sesquiterpenes) resulted in reduced visitation. This demonstrates that stingless bees are capable of learning complex scents and responding to multiple compounds within the resin bouquet, and may be more selective for resin sources than floral sources [16,23].

After locating a resin source, stingless bees use their mandibles to gather resin from plant buds, leaves, flowers, or bark. They then use the tarsi and basitarsi on their front and middle legs to load this sticky material onto their corbiculae [55,88] (see Supplementary Materials). They repeat this process until they have amassed a sizable resin load, which they carry back to the nest. Some stingless bees induce plants to secrete resin by biting plant tissues and collecting the resin that seeps from the resulting wound [47,91]. Howard [65] reported that foragers of certain species can milk an active resin source for days or weeks at a time. In fact, since resin foraging can damage tissues, some stingless bees (e.g., *Trigona fuscipennis* and *Trigona nigerrima*) have been considered pests for agricultural crops [92]. Many stingless bee species collect resin in groups and vigorously defend preferred resin resources. Some species have been observed fighting to the death over resin, stealing cerumen from other nests, or harvesting materials from abandoned nests [62,65]. Howard [65] suggested that these behaviors indicate that resin is a precious resource for many stingless bee species, and that resin resource availability is likely a limiting factor for colony growth.

Stingless bees demonstrate clear preferences for some resin-producing plants, and neglect others [15,24,62]. The factors that determine stingless bees’ resin preferences are unknown. As discussed, bees may target certain plants based on the potency of the antimicrobial or repellent properties their resins possess [15]. Morphological parameters likely also dictate the resources that each species can access. Some minute species (e.g., *Trigona jatiformis*) seek out resin sources that are too small to be seen with the naked eye; the resin they collect is only identifiable once it has been accumulated in the bees’ corbiculae [65]. Larger bees likely neglect minute resin sources, but may be more likely to use their mandibles to induce plant injury to encourage resin flow. Smaller bees often take advantage of the resin sources tapped by larger species, either collecting alongside the larger bees, attempting to supplant them, or waiting until the larger bees have abandoned the resin source [65]. So far, these behaviors have been examined through the lens of competition, but the interdependence implicit in these interactions is both fascinating and noteworthy. The fact that certain bee species depend on other bee species for access to resin resources could have implications for stingless bee conservation.

### 3.4. Resin Handling

Once resin foragers return to the hive, they unload resin from their corbiculae on their own, or with the help of another worker [50,55,88]. The often brightly colored resin loads are mixed with wax to form cerumen, or incorporated into other nest structures. The terpenoid compounds in resin become oxidized over time, causing them to darken in color [93]. Unloading and processing resin is a laborious task; bees must be careful to manipulate this material without getting stuck. In one study, it took *Plebeia lucii* and *Frieseomelitta varia* foragers seven to thirteen minutes to unload resin back at the hive. Most of this time was spent removing resin residue from their tarsi [55].

How do stingless bees handle the sticky substance that they use to immobilize and kill their enemies, without harming themselves? Stingless bee body parts do not appear to possess inherently anti-adhesive properties. Gastauer et al. [94] used electron microscopy and adhesive force experiments to compare the mandibles of stingless bee *Tetragonisca angustula* and the trochanter of invader ant *Camponotus sericeiventris*. They determined that resin actually adheres more to the smooth bee mandible than it does to the scaled ant trochanter. This suggests that stingless bees must utilize a lubricating substance (e.g., secretions or nectar) to reduce adhesion of resin to mandibles.

Some studies suggest that the ability to produce lubricating substances and avoid adhesive hazards is associated with bee age and physiological development. In *T. angustula, Plebeia emerina,* and *Trigona (Hypotrigona) grihodoi,* resin handling is a task reserved for advanced-age workers [49,88,95]. An examination of the head salivary and intramandibular gland morphology of *P. emerina* suggested that when workers reach a certain developmental stage, they begin to produce secretions that help maintain propolis viscosity and allow the bees to handle this material without getting stuck [49]. The development of the head salivary and intramandibular glands late in life does not occur in all stingless bee species, and may occur only in bees that maintain viscous propolis stores or deposit resins within the hive.

There is some evidence for a genetic basis for “propolis preparation”—presumably resin handling—in *Melipona quadrifasciata* [96]. In one study, young bees (1–5 days old) from ten different source colonies were tagged and introduced into three different observation hives, with each observation hive containing bees from all ten source colonies. Their activities were observed for 35 days. Resin foraging was similar across source colonies, but bees from certain source colonies were significantly more prone to participate in propolis preparation [96]. This study was limited in that observation colonies were made up of workers from a single age cohort. Stingless bee workers demonstrate plasticity, with workers changing tasks based on the needs of the colony. In several species, resin handling occurs late in life, so the lack of older bees in observation hives may have influenced the resin handling behavior of the young bees in this experiment. If this is the case, the higher incidence of propolis preparation observed in bees from certain source colonies may indicate higher levels of plasticity, and not necessarily a genetic predisposition to resin handling, but this possibility warrants further investigation.

### 3.5. Resin Shapes the Cuticular Chemical Profile of Some Stingless Bees

For some stingless bee species, the presence of resin inside the nest space can also influence the physical properties of the bees themselves. The outer layer of the insect cuticle is made up of lipid compounds that serve a variety of functions. These compounds help protect insects from predators, desiccation, and abrasion, and they also play a role in nestmate recognition and other forms of communication [97]. The so-called cuticular chemical profile consists of compounds that are polar (e.g., alcohols, esters, ketones, aldehydes, and oxidized terpenes) and non-polar (e.g., n-alkanes, alkenes, and methyl-branched alkanes) [98]. These compounds can be self-produced or environmentally acquired [74]. Some stingless bee species acquire certain cuticular compounds (e.g., terpenoids, such as such as mono-, sesqui- and triterpenes) from resin [93,98,99,100,101].

Among social insects, stingless bees are the only group known to enrich their cuticular chemical profile with resin-derived compounds [16,74]. To our knowledge, this has not been examined in *A. mellifera*. This trait appears to have emerged separately in multiple stingless bee lineages. It occurs in more evolutionarily derived genera and is generally absent from more basal genera (e.g., *Melipona* and *Plebeia*), with at least one exception [102,103]. Despite overlap in foraging behavior (i.e., different species often utilize many of the same resin sources) the uptake of resin-derived compounds results in species-specific terpenoid profiles that are consistent across diverse geographic regions [65,98,99,103,104]. The overlap between the nest entrance chemical profile and the cuticular chemical profile of multiple stingless bee species suggests that these compounds are most likely derived from the resin present in the nest environment [99,104]. Leonhardt et al. [104] suggested that some kind of filter mechanism must enable the uptake of certain compounds while excluding others. Resin collected from the corbiculae of stingless bees is not chemically different from resin collected by researchers directly at resin wounds. Thus, if resin is modified, this must occur within the hive [104]. Different stingless bee species may possess different enzymes or microbial associates that alter the incoming resins, resulting in the species-specific selective uptake of terpenoid compounds. Additionally, or alternatively, genetically determined species-specific differences in cuticular chemistry could determine which compounds ‘bind’ to the bee [104]. Further research is needed to understand how and why certain resin-derived compounds enrich the cuticular chemical profile of certain stingless bee species, and to further elucidate the implications this has for colony function.

Recent studies have demonstrated that a resin-enriched cuticular chemical profile can help protect bees from predators and may reduce interspecific aggression, facilitating nest aggregations. Resin confers repellant properties to the cuticle of some stingless bee species, adding to the effects of the genetically determined repellent compounds that the bees produce themselves [74]. The repellent properties of cuticular terpenoids were observed in a study that compared two species—*Tetragonula carbonaria*, a bee that collects extensive amounts of resin, whose cuticular compounds are 50% resin-derived, and *Austroplebeia australis*, a bee that collects minimal resin, whose cuticular compounds are just 1% resin-derived. In behavioral assays, high-resin *T. carbonaria* bees repelled predator ants, but low-resin *A. australis* did not. Washing both bee species diminished the ants’ preference, suggesting that repellant properties can be attributed to the resin-derived compounds found on the bees’ cuticle [74].

Resin-derived terpenes present in the cuticle might also help facilitate nest aggregations. These compounds may mask chemical differences between bee species, contributing to reduced interspecific aggression [105]. One study compared aggressive behaviors between bees from the same nest aggregation, different aggregations, and non-aggregated nests, and found that aggression was reduced between bees from associated colonies [100]. The authors hypothesized that the presence of resin-derived terpenoids, specifically sesquiterpenes, mediates reduced aggression. They experimentally manipulated the chemical profile of *Tetragonula melanocephala*, a stingless bee whose cuticle lacks sesquiterpenes, and found that applying either pure sesquiterpenes or an extract derived from the sesquiterpene-rich cuticle of *Tetragonula collina* (an unusually peaceable bee) to non-nestmates resulted in decreased aggression. Based on these results, the authors suggested that sesquiterpenes may facilitate nesting aggregations in tropical environments, but more research is needed to determine the precise role that resin-derived compounds play in mediating complex inter- and intraspecies interactions.

While it is clear that resin-derived compounds contribute to the cuticular chemical profile of many stingless bee species, and this profile is thought to influence nestmate recognition [106,107], the impact of resin on nestmate recognition is less clear. When Jones et al. [75] exposed *Tetragonisca angustula* workers to extracts made from nestmate and non-nestmate resin or wax, all treatments resulted in decreased acceptance rates, regardless of the material source. In the same study, Jones et al. [75] transferred resin stores from donor colonies to recipient colonies to determine whether bees use in-hive resin stores as a reference for recognition cues. They observed decreased acceptance of nestmates in donor colonies after interference, but no change in non-nestmate acceptance by donors. They also observed increased acceptance of non-nestmates in recipient colonies, and general guard confusion. Based on these results, the authors concluded that wax and resin do not contribute to nestmate recognition in *T. angustula*. However, it is possible that the artificial transfer of resin-derived compounds (i.e., exposing bees to resin-enriched hexane extract rather than raw wax or resin) impacted these results. Similarly, conducting behavioral assays only a short time after transferring resin stores from donor colonies to recipient colonies could have led to increased defensive behavior, muddling the nestmate recognition findings. Further studies are needed to determine the potential relationship between nest materials and nestmate recognition in stingless bees.

### 3.6. Microbiota Associated with Stingless Bees

There is growing interest in sequencing and understanding the functional significance of the microbial communities associated with stingless bees and their nests [108,109,110,111,112,113]. The antimicrobial activity of stingless bee resin has been studied extensively in a human health context [9,114,115,116,117,118], and much stingless bee research mentions, in passing, that resin likely plays a role in shaping the microbiota inside the nest. However, despite the demonstrated importance of bacteria and fungi to stingless bee colony function [17,18,119], and the assumed importance of resin in maintaining microbial balance [9,29], whether and how resin modulates the microbial communities associated with stingless bees and their nest spaces is understudied.

Recent studies provide some insight into these complex interactions. In *A. mellifera* colonies, researchers have found that the presence of a propolis envelope stabilizes the microbial communities found in bees’ guts and on the cuticle of their mouthparts. The propolis envelope is thought to support the proliferation of putatively beneficial bacterial associates, and reduce the expression of pathogenic or opportunistic microbes [19,20]. However, the role of resin in shaping the microbiota associated with stingless bees (e.g., cuticular, gut, whole-bee, and nest microbiomes) is less clear.

One recent study compared the bacterial communities associated with the interior nest surfaces of four stingless bee species (*Frieseomelitta varia, Melipona quadrifasciata*, *Tetragonisca angustula,* and *Trigona spinipes*) [113]. Differences in these bacterial communities were attributed, in part, to the diverse materials that each species uses in nest construction (e.g., clay, resin, wax, and feces). Unfortunately, this study did not include a detailed characterization of nest architecture for the species in question, and it is unclear which surfaces were swabbed for bacteria. Nevertheless, this study points toward the importance of understanding the complex microbial ecosystems that exist within the stingless bee nest, and the broader role of nest construction materials, such as resin, in shaping those ecosystems.

Leonhardt and Kaltenpoth [110] used sequencing to characterize the microbiota associated with three sympatric Australian stingless bee species, two that incorporate large quantities of resin in their nest (*Tetragonula carbonaria* and *Trigona hockingsii*), and one that uses almost no resin (*Austroplebeia australis*). DNA was extracted from six worker bees from ten different colonies, and whole-bee microbiomes were compared. Species-specific differences in microbial communities were observed. However, more species must be sampled to determine whether these changes can be attributed to the presence or absence of resin. Moreover, since many additional species-specific factors (e.g., genotype, diet, external environment, and nest construction materials) can influence microbial communities [113,120], within species comparisons (i.e., comparing the microbial communities associated with high-resin colonies vs. low-resin colonies, as occurred in *A. mellifera* studies) may be instructive.

Another study examined the rate of mold growth on the bodies of some of the same high-resin (*T. carbonaria*) and low-resin (*A. australis*) bees, to determine whether a resin-rich environment confers antimicrobial properties to the stingless bee cuticle [74]. In this study, the rate of mold growth was not found to differ between species. However, it is possible that resin-poor *A. australis* has evolved compensatory physiological traits (e.g., increased secretion of self-produced antimicrobials) to replace resin resources, as proposed by Roubik [29]. If this is the case, the cuticle of both the resin-rich and resin-poor species should possess antimicrobial compounds that inhibit the growth of mold, with the difference being the source (self-produced versus environmentally acquired). Further studies are needed to elucidate the impact of resin on the bacteria and fungi naturally present on the stingless bee cuticle and within the stingless bee nest, and to determine how the presence of resin relates to self-produced antimicrobial compounds.

Perhaps the most compelling example of the importance of microbial associates to colony function is the mutualism between the stingless bee *Scaptotrigona depilis* and a fungus of the genus *Zygosaccharomyces* [17,18]. This fungus exists in a dormant state in the cerumen of *S. depilis* colonies. When it comes into contact with the liquid larval food found inside the brood cells, it enters a growth phase, extending visible white mycelia from the brood cell wall towards the larval food supply. Originally thought to be pathogenic, these mycelia actually produce steroid precursors that *S. depilis* larvae require for pupation [17]. Since resin is a key ingredient in cerumen and since it inhibits the growth of some but not all microbes, it is likely that the presence of resin helps support the growth of *Zygosaccharomyces*. This mutualism is just one visible example of countless probable stingless bee-microbe associations that could prove essential to colony function.

Though further research is needed, this evidence, alongside recent discoveries demonstrating that propolis helps shape the gut and mouthpart microbiomes of *A. mellifera*, suggests that resin may help stabilize and/or support microbial communities that could prove essential to stingless bee colony function.

## 4. Future Studies

Existing research demonstrates that resin use is vital to stingless bee colony function. Resin is essential to nest construction and defense, and many species invest substantial effort in resin foraging and handling. Resin-derived compounds influence the cuticular chemical profiles of many stingless bee species, and resin likely shapes the microbial communities associated with stingless bees and their nests. Further research is needed in each of these individual areas, and at their intersections.

What are the causes and consequences of different levels of resin use by different species, and by different colonies of the same species? Some stingless bees invest vast amounts of energy in resin collection, some collect only the minimum necessary to build nest structures, and there is at least one example of a stingless bee that does not use resin at all [44]. Differences in resin use can occur even when many major variables (e.g., species, location, and hive structure) remain constant. Roubik [10] attributed individual variation in nest architecture—including the thickness of the resinous batumen surrounding the nest—to three possible causes: (1) nest age, (2) bee genetics, and (3) micro-environment (e.g., predators, parasites, symbionts, rain, wind, and sun). Further studies are needed to evaluate the effects of each of these factors on resin use, and to determine how differences in resin use impact colony function, cuticular chemical profile, and the microbial communities associated with stingless bees. For example, if resin contributes significantly to colony function, do low-resin species or low-resin colonies compensate physiologically for the lack of resin in their space (e.g., through increased antimicrobial secretions or increased diversification of self-produced cuticular chemical compounds) [29]? More broadly, might examining tradeoffs between the secretion of antimicrobial compounds and the collection of antimicrobial materials help inform our understanding of the evolution of social insects? For example, is it possible that intensive resin use emerged in stingless bees following the loss of the stinging apparatus, and the antimicrobial venom that may have accompanied it [38]? Mixing secreted and collected materials for nest construction is common in invertebrates, but the selective pressures that favor secretion versus collection are poorly understood, and have not been examined in stingless bees [121].

How does the presence and prevalence of resin in the nest space impact other aspects of the stingless bee nest ecosystem and colony function? Since resin is present throughout the nest in the form of cerumen and is in direct contact with both brood and food stores, it is possible that resin-derived compounds may leech into stingless bee honey and pollen, enriching these food sources with phytochemicals [16]. Honey produced by *A. mellifera* has been found to contain phytochemicals that likely originate from propolis [36]. Does resin also contribute phytochemicals to stingless bee honey? Does the amount of resin that bees incorporate in the nest environment affect the quantity or type of resin-derived compounds found in the honey? If resin-derived phytochemicals are an important part of the stingless bee diet, then how might certain beekeeping practices (e.g., introducing sugar syrup or *A. mellifera* honey in periods of dearth, or removing excess resin stores from a colony to facilitate colony management) impact stingless bee health?

As previously mentioned, the microbiota associated with stingless bees and their nests is a vast and fascinating area, the advancement of which could inform questions relating to the chemical ecology of stingless bees, among other aspects of colony function. For example, do the resin-derived compounds that comprise the cuticular chemical profiles of some stingless bee species impact their cuticular microbiome? How does this affect the ability of the cuticular chemical profile to repel predators, reduce aggression, etc.? Does the cuticular microbiome, in turn, influence the cuticular chemical profile? Does resin help shape the microbiota associated with stingless bees and their nests? How does this impact colony function?

There are interesting points of overlap—and important differences—in resin use by honey bees and stingless bees. These points of comparison could inform future research in both study systems. For instance, does resin use constitute a social immunity mechanism in stingless bees [28]? Does resin use inhibit the growth of microbial pathogens within the stingless bee nest space? Does the presence of resin help modulate the stingless bee immune system, as occurs in *A. mellifera*? If so, how does immune expression compare in high-resin and low-resin species? Future stingless bee research could draw from recent honey bee research [31,32] to investigate these questions. In turn, honey bee research could draw from stingless bee research to investigate, for example, whether *A. mellifera* foragers use olfactory cues to locate resin sources, as occurs in stingless bees, and whether the presence of a propolis envelope influences the cuticular chemical profile of *A. mellifera*.

Underlying all of these questions is the need for more research on the natural history of stingless bees. Many of the predominant natural history studies in this field date back over half a century and cover a relatively small number of species. While informative, these studies cannot be considered representative because stingless bees are so diverse, and resin use is such a variable trait. More research is needed to add further breadth to the foundational studies that figure so strongly into current conceptions of resin use in stingless bees. In this pursuit, and in other areas of future research, there is an important opportunity to partner with and follow the leadership of the stingless beekeepers, indigenous communities, and stewards of local ecological knowledge that have been in relationship with stingless bees for generations.

## 5. Conclusions

Understanding the role of resin use in stingless bee colony function grows increasingly urgent as changes in beekeeping and land use practices occur, potentially diminishing stingless bees’ ability to incorporate resin into their nest environment [3,4,15]. In recent decades, the massification of beekeeping operations and the transportation of stingless bee colonies to monocrop fields for pollination services has expanded [122], and—among other deleterious effects—these changes could limit bees’ access to diverse resin sources, potentially inhibiting nest construction and defense and influencing their cuticular chemical profiles [123] and microbial associates [124]. Bees are already known to substitute resin for human-made products such as wet paint, adhesives, and asphalt [10,125]. As the role of resin likely extends beyond its adhesive properties, the extent to which such substitutions—potentially driven by resin resource scarcity—impact stingless bee colony function in the long term is cause for concern.

A deeper understanding of the importance of resin use for stingless bee colony function could lend support to the conservation of resin-rich non-floral resources that might otherwise be overlooked [43]. Since bees depend on diverse resin sources to carry out a variety of functions, targeted conservation efforts could bolster stingless bees’ ability to defend against pathogens, parasites, and predators, and support colony health in ways we cannot yet anticipate [15]. In this context, it is crucial to review and expand upon the many varied studies of resin use in stingless bees so we can understand and appreciate its importance for colony function and stingless bee health.

## Figures and Tables

**Table 1:** Resin-rich materials and nest structures.

Cerumen	A mixture of wax and resin that stingless bees use to build brood combs, honey and pollen pots, and other nest structures
Deposit-resins	Caches of resin stored by some species on the floor or walls of their nests (also known as resin deposits or viscous propolis deposits)
Propolis	Resin mixed with small amounts of salivary gland secretions and wax and used to seal cracks and crevices throughout the nest
Geopropolis	Resin mixed with soil, silt, and/or sand particles
Batumen	A wall-like structure that often contains resin; many species build a batumen to separate the inner nest environment from the external world
Nest entrances	For some species, nest entrances consist of hardened resin tubes, which can extend both inside and outside the nest

## Supplementary Materials

The following are available online at https://www.mdpi.com/article/10.3390/insects12080719/s1, Video S1: *Scaptotrigona mexicana* forager collecting large resin load.
